# Profile of mood states-27: A valid and reliable measure of negative and positive affect for brazilian pregnant women

**DOI:** 10.1192/j.eurpsy.2021.1036

**Published:** 2021-08-13

**Authors:** M. Barros, M. Aguiar, A. Macedo, A.T. Pereira

**Affiliations:** 1 Departamento De Ciências Naturais, Universidade do Sudoeste da Bahia - UESB, Vitória da Conquista, Bahia, Brazil; 2 Instituto De Psicologia Médica, Universidade de Coimbra, Coimbra, Portugal; 3 Pos Graduação Em Psicologia Da Saúde, Universidade Federal da Bahia, Vitória da Conquista, Brazil; 4 Institute Of Psychological Medicine, Faculty Of Medicine, University of Coimbra, Coimbra, Portugal; 5 Institute Of Psychological Medicine, Faculty Of Medicine, University of Coimbra, coimbra, Portugal

**Keywords:** negative affect, Depression, validation, pregnancy

## Abstract

**Introduction:**

The original Profile of Mood States (McNair et al. 1971) is composed of 65 items to measure six affect dimensions. There is a Portuguese shorter version (POMS-27) for the perinatal period (Bos et al. 2013), which reduced the number of items (adjectives) down to 27. Although briefer, it still covers relevant mood states with good psychometric properties, being considered a good alternative to use during this demanding and busy period.

**Objectives:**

To test if PoMS-27, composed of four dimensions (Depression, Anxiety, Vigour, Fatigue) and one second-order factor (Negative Affect, composed by the former two) is valid and reliable to be used with pregnant women from Brazil.

**Methods:**

After confirming the items semantic equivalence and slightly adapt two adjectives from European to Brazilian Portuguese, 350 pregnant women (Mean age: 30.01±5.452; Mean gestation weeks=25.17±6.55), with uncomplicated pregnancies, completed the PoMS-27 and the Brazilian recently validated versions of Perinatal Depression Screening Scale and Perinatal Anxiety Screening Scale (PDSS; PASS; Barros et al. 2021). SPSS and AMOS software were used.

**Results:**

After deleting two items (20 and 22) and some errors were correlated, CFA indicated that the PoMS-25 four-dimensional model presented an acceptable fit (χ2=3.163; RMSEA=.079, CFI=.868, TLI=.849). The Cronbach alphas of the dimensions were: Depression (.82), Anxiety (.86), Negative Affect (.89), Fatigue (.80) and Vigor (.50).

**Conclusions:**

Within this psychometric study we found useful, valid and reliable measure of negative and positive affect for Brazilian pregnant women. From now on we will denominate it Profile of Mood States-25Br

